# Chronic osteomyelitis with pathological fracture induced by *Mycoplasma hominis* infection: a case report and review of the literature

**DOI:** 10.3389/fmed.2025.1510753

**Published:** 2025-01-27

**Authors:** Ling-Na Zhu, Wen-Bin Shen, Xiao-Yan Zou, Jiang-Cheng Zuo, Ning Xiao

**Affiliations:** Yiling People’s Hospital of Yichang City, Yichang, Hubei, China

**Keywords:** *Mycoplasma hominis*, chronic osteomyelitis, pathological fracture, mass spectrometry, sinus tract

## Abstract

*Mycoplasma hominis*, commonly residing in the genitourinary tract, can cause rare extragenital infections, especially in immunocompromised individuals. This report details a case of chronic osteomyelitis with a pathological femur fracture in a 79-year-old woman. Despite a history of bone tuberculosis, the infection was identified as *Mycoplasma hominis* through culture and mass spectrometry, highlighting the diagnostic challenges due to the organism’s lack of a cell wall. This case underscores the necessity for advanced diagnostic methods and awareness of *Mycoplasma hominis* in non-urogenital infections.

## 1 Introduction

*Mycoplasma hominis* is a bacterial species belonging to the *Mycoplasma* genus (1). Notably, It is capable of invading and residing within human cells (2). Together with ureaplasmas, mycoplasmas represent the smallest known free-living organisms. Lacking a cell wall, these bacteria do not retain Gram stain, making traditional Gram staining techniques ineffective. Clinically, *Mycoplasma hominis* has been linked to conditions such as pelvic inflammatory disease and bacterial vaginosis (3, 4), as well as contributing to male infertility (5). It is recognized as a causative agent of sexually transmitted infections (6) and can be effectively treated with clindamycin (7). Additionally, this microorganism may establish latent infections in the chorionic villi of pregnant women, potentially influencing pregnancy outcomes (8).

The extragenital presence of *Mycoplasma hominis* is uncommon, and it is thought that the immune status of the host may contribute as a predisposing factor (9). This report describes a rare case of chronic osteomyelitis with a pathological fracture caused by *Mycoplasma hominis* infection.

## 2 Case

**FIGURE 1:**
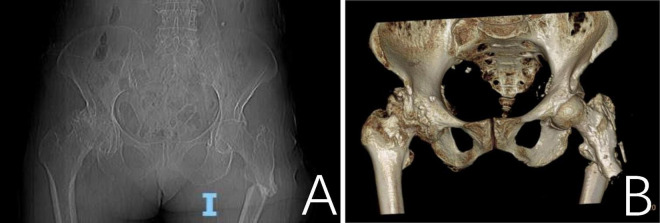
A CT scan revealed a fracture of the proximal segment of the left femur with misalignment and overlapping of the fracture ends. **(A)** Thickening of the cortical bone in the mid-to-proximal segment of the left femur with irregular margins; a linear, bone-like hyperdense shadow is observed laterally. The medullary cavity shows unevenly increased bone density. **(B)** The three-dimensional CT reconstruction of the proximal left femur demonstrating fracture morphology.

A 79-year-old female patient presented with a left hip injury following a fall, accompanied by severe pain at the site of injury and difficulty with movement. The patient reported that 1 month ago, without any obvious inciting factors, she developed pain in her left thigh, along with ulceration and exudation on the posterior aspect of the thigh. No medical treatment was sought at that time. Her medical history includes a diagnosis of “bone tuberculosis” 50 years ago, which improved with consistent anti-tuberculosis therapy. Physical examination revealed significant deep tenderness in the left inguinal region, along with notable crepitus and abnormal mobility of the left hip joint. Two old sinus scars were observed on the lateral aspect of the left thigh, along with a 0.5 cm diameter unhealed sinus on the posterior thigh, which showed slight exudation. The skin over the affected area was erythematous and swollen. Range of motion in the hip joint was restricted, and the left lower limb demonstrated marked external rotation, adduction deformity, and a shortening of approximately 2 cm. Peripheral circulation in the affected limb remained intact. A CT scan confirmed a left proximal femur fracture with displaced and overlapping fracture fragments. The middle and upper cortices of the left femur showed thickening, irregular margins, and increased strip-like bone density on the lateral side, along with unevenly increased density in the medullary cavity (Figure 1).

Laboratory findings revealed elevated C-reactive protein (CRP) at 6.14 mg/L, hypokalemia with serum potassium at 3.10 mmol/L, and hypoalbuminemia with albumin levels at 39.3 g/L. Sinus exudate was sent for culture and sensitivity testing. The patient received debridement, dressing changes, and intravenous antibiotic therapy with Cefazolin (1 g IV daily for 7 days).

**FIGURE 2:**
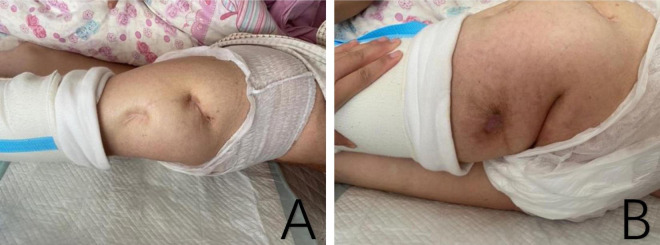
The pictures showed the healed sinus tract on the lateral and posterior proximal femur, with no obvious redness or swelling. **(A)** Lateral proximal of left femur. **(B)** Posterior proximal of left femur.

One month after discharge, we followed up with the patient. Her vital signs were stable. There was significant improvement in the swelling and pain of the left hip, although the hip joint deformity persisted with mild tenderness. The sinus tract on the posterior thigh had healed completely (Figure 2). The patient had received a 1-week course of intravenous penicillin at a local clinic following discharge and did not report the use of any additional antimicrobial agents.

## 3 Methods

Tuberculosis smear, tuberculosis culture and *Mycobacterium tuberculosis* nucleic acid detection were performed on the sinus secretions, and general bacterial smear and general bacterial culture and identification were performed simultaneously.

Mass spectrometry was employed for further detection:

We collected a sample from the deep secretion of the patient’s sinus for testing. The specimen was inoculated onto two blood agar plates, and after 72 h of incubation, pinpoint like colonies were observed. The sample was then subjected to mass spectrometry. After the initial identification, another sample was collected for testing, again. The specimen was inoculated onto a blood agar plate for 48 h to pinpoint like colonies appeared. A single colony was then subcultured onto a new blood agar plate for pure culture. After 72 h, the colonies were identified using mass spectrometry. The two tests produced identical results, indicating the presence of *Mycoplasma hominis*.

Equipment: The mass spectrometry identification instrument was the EXS-3000 fully automated microbial mass spectrometer from Chongqing *Zhongyuan^®^*.

Reagents: Columbia blood agar plates were from Zhengzhou Antu Bioengineering Co., Ltd. (No. 20172400631), fluorescence staining reagents were from Zhuhai Beso Biotechnology Co., Ltd. (No. C112201), Mycoplasma culture identification and sensitivity reagent kits from Zhengzhou *Antu^®^* Bioengineering Co., Ltd. (No. 20160058).

Principle: A known strains database was established for microbial protein profile detection. The differences in ribosomal proteins (2–20 kDa) among different strains were compared to the reference profiles in the database to obtain identification results. The procedure involved smearing a small, fresh, pure colony onto a target plate in a clockwise direction to form a thin film, which is dried, then adding 1 μL of 70% formic acid solution. After drying, 1 μL of matrix solution is added, dried, and subjected to mass spectrometry identification.

A mycoplasma IES (Immunoenzymatic Screening) kit was applied:

The colonies cultured on blood agar for 3 days were tested using the mycoplasma kit. The kit was based on cultivation and biochemical reactions. After Mycoplasma had been cultivated, arginine can be decomposed by arginase in *Mycoplasma hominis* and release NH3 (10, 11), which increases the pH of the culture medium. The result was determined based on the color change indicated by a pH indicator. The antibiotic susceptibility test plate was coated with 12 different antibiotics. If the tested Mycoplasma was sensitive to the coated antibiotics, its enzymatic activity was inhibited, resulting in no color change.

## 4 Discussion

**FIGURE 3:**
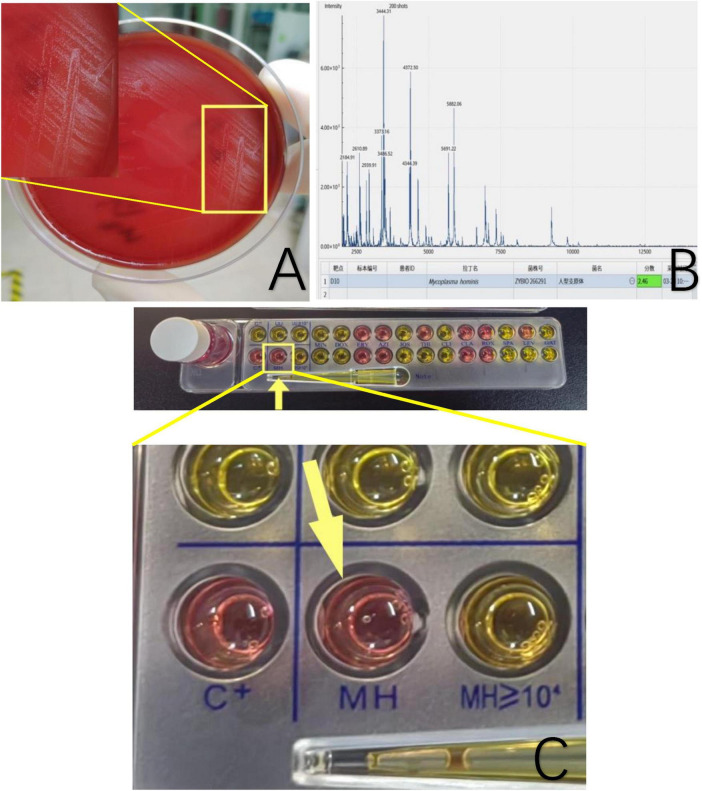
**(A)** Sinus secretion culture of the patient’s femur shows pinpoint grayish-white raised colonies. **(B)** Mass spectrometry identification confirmed *Mycoplasma hominis* with a score of 2.46. The software features dual functions of spectrum acquisition and microbial identification. After acquiring the mass spectrum, the obtained spectrum is matched and searched against a standard database to complete the microbial identification of the sample. The software directly displays the identification results of the strain, with color coding to indicate the confidence level of the identification at each sample site. The software uses a scoring system based on a three-point scale. When the score is ≥2.0, the identification result is displayed in green, indicating a possible species-level identification, with higher scores reflecting greater confidence in the species-level identification. When the score is ≥1.7 but <2.0, the result is displayed in yellow, indicating a possible genus-level identification, with higher scores reflecting greater confidence in the genus-level identification. **(C)** A mycoplasma IES kit was applied. The color turns from orange to red or peachblow implicating the growth of *Mycoplasma hominis*.

The cultures of sinus tract discharge for *Mycobacterium tuberculosis* were repeated three times, and all results were negative, thus excluding tuberculosis infection. General bacterial smears and cultures also showed no bacteria. On the third day of culture, pinpoint-like colonies appeared, and further culture and observation continued (Figure 3A). The score for mass spectrometry of the sample is 2.46. Based on the colony morphology and Mycoplasma culture reactions, *Mycoplasma hominis* were confirmed (Figure 3B). Furthermore, the Mycoplasma IES kit was applied to confirm *Mycoplasma hominis* infection, we observed that the reagent of the color turned from orange to red implicating the growth of *Mycoplasma hominis* (Figure 3C).

Mycoplasmas are common commensals in the oropharynx and genital tract. Of the 18 species in humans, only a few are pathogenic, including *Ureaplasma urealyticum*, *Ureaplasma parvum*, *Mycoplasma hominis* and *Mycoplasma genitalium*, which are linked to genitourinary tract infections (12). *Mycoplasma hominis* and *Ureaplasma urealyticum* are commonly found in the lower urogenital tract of healthy adults, with colonization associated with age, socioeconomic status, multiple sexual partners, ethnicity, hormonal status, and pregnancy (13, 14). *Mycoplasma hominis* is present in approximately 10% of healthy women, while *Ureaplasma urealyticum* can be detected in up to 50% (13). Additionally, *Mycoplasma genitalium* has been detected in the genital tract of asymptomatic individuals (15).

*Mycoplasma hominis* with their prevalence shows an upward trend (16, 17). *Mycoplasma hominis* is a cell wall-deficient bacterium belonging to the class Mollicutes (18). It commonly colonizes the human urogenital tract as significant pathogens responsible for nongonococcal urethritis, cervicitis, pelvic inflammatory disease, orchitis, and epididymitis, potentially leading to infertility in both sexes (19). As genital mycoplasmas, *Mycoplasma hominis* predominantly causes mucosa-associated urogenital infections, while non-urogenital infections are relatively uncommon (17, 20). While *Mycoplasma hominis* primarily colonizes the urogenital tract, its role in non-genital infections, particularly in immunocompromised patients (21–24), is increasingly recognized. Previous studies have identified *Mycoplasma hominis* as a causative agent in various non-genital infections, including osteomyelitis (25, 26). Stabler et al. described a case involving rectal abscesses attributed to *Mycoplasma hominis* infection (9). Similarly, Hulme-Jones et al. documented a trochanteric bursal infection in a post-pancreas-kidney transplant patient caused by *Mycoplasma hominis* (27). Castillo et al. reported a case of limb soft tissue infection linked to this pathogen (28). Additionally, Bethel et al. detailed a postoperative pelvic fracture-associated *Mycoplasma hominis* infection in a trauma patient (29).

We reviewed a selection of case reports of extragenital wound infections caused by *Mycoplasma hominis* over the past 30 years (Table 1), which predominantly affected middle-aged and elderly males (approximately 80%, with 15 out of 19 cases involving males aged ≥ 40 years). The reported cases involved various surgical and trauma-related infection sites, including sternotomy, vascular grafts, spinal surgeries, joint replacements, and mediastinal regions. The most commonly used antibiotics were tetracyclines (e.g., doxycycline and minocycline), often combined with other agents such as clindamycin, levofloxacin, or rifampicin. In recent years, newer antibiotic regimens, such as tigecycline, have been increasingly employed, particularly in complex or resistant infections. While most patients experienced favorable outcomes (approximately 95%, with 18 out of 19 cases resulting in successful healing), one fatal case was reported due to complications of mediastinitis and pleuritis.

**TABLE 1:** A selection of case reports of extragenital wound infections caused by *Mycoplasma hominis* (1994–2024).

References	Age (yrs), sex	Organism	Cite of infection	Antibiotics	Out-comes
Pigrau et al. (31)	63, male	*M. h.* *U.u.*	Sternotomy	Clindamycin, Gentamicin, Doxycycline	Healed
Sielaff et al. (47)	62, male 63, male 48, male	*M.h.*	Cardiovascular surgery	Doxycycline, Clindamycin	Healed
Mossad et al. (48)	–	*M.h.*	Sternotomy	Clindamycin, Doxycycline	Healed
Vogel et al. (49)	48, male 29, female	*M.h.*	Liver transplantation	Doxycycline, Clindamycin	Healed
Hopkins et al. (50)	55, male 52, male 21, male 17, male	*M.h.*	Heart and lung transplantation	Doxycycline Ciprofloxacin Clindamycin	Healed
Krijnen et al. (30)	11, female	*M.h.*	Spine	Doxycycline	Healed
Garcia-de-la-Fuente et al. (32)	77, male	*M.h.* *U.u.*	Sternotomy	Doxycycline, Clindamycin	Dead
Marini et al. (51)	73, male	*M.h.*	Vascular allograft (superficial femoral)	Rifampicin, Amoxicillin/ Clavulanic Acid, Fluoroquinolones	Healed
Lee et al. (52)	71, male 76, male	*M.h.*	TKA	Erythromycin, Azithromycin, Clarythromycin	Healed
Myers et al. (53)	55, male	*M.h.*	Ascending aortic	Doxycycline, Moxifloxacine	Healed
Yamakami (54)	28, female	*M.h.*	Lower leg ulceration		Healed
Flouzat-Lachaniette et al. (55)	38, female	*M.h.*	Total disk replacement	Doxycycline	Healed
Wynes et al. (56)	33, male	*M.h.*	Subtalar joint	Levofloxacin, Doxycycline	Healed
Whitson et al. (57)	17, male	*M.h.*	Spinal cord	Vancomycin, Moxifloxacin, Doxycycline	healed
Antonic et.al (58)	63, male	*M.h.*	Ascending aorta	Tigecycline	Healed
Ng et al. (59)	63, male	*M.h.*	Lumbar	Doxycycline	Healed
Qamar et al. (60)	25, female	*M.h.*	Intracranial	Levofloxacin, Doxycycline Daptomycin	Healed
Kitagawa et al. (35)	54, male	*M.h.*	Posterior mediastinum (cardiac surgery)	Minocycline	Healed
Huang et al. (61)	43, male	*M.h.* *P.a.*	Right pelvic wounds (trauma)	Minocycline, Meropenem, Teicoplanin	Healed
Kuo et al. (62)	75, male	*M.h.*	Left lower leg	Levofoxacin	Healed

M.h., *Mycoplasma hominis*; U.u., *Ureaplasma urealyticum*; P.a., *Pseudomonas aeruginos*.

*Mycoplasma hominis*, in some instances, co-infections with *Ureaplasma urealyticum* predominantly affect immunocompromised or post-surgical patients, highlighting specific susceptibility patterns. Patients undergoing major surgical procedures, such as sternotomy, vascular grafting, or organ transplantation, appear to be at higher risk due to the invasive nature of the interventions and associated immunosuppressive therapies. Moreover, certain anatomical regions, such as the mediastinum, pelvis, and spine, may provide an environment conducive to the growth of *Mycoplasma hominis.*

Age and gender also appear to play a role in susceptibility. Most cases involved middle-aged to elderly males, potentially due to higher rates of comorbidities such as diabetes, cardiovascular disease, or previous surgeries in this population. Younger patients, although less frequently affected, were often associated with specific risk factors such as trauma or rare predisposing conditions (e.g., scoliosis surgery in an 11-year-old female patient) (30). Additionally, co-infections with *Ureaplasma urealyticum* suggest that these pathogens may act synergistically to exploit host vulnerabilities, as seen in the fatal case of mediastinitis complicated by pleuritis and pericarditis (31, 32). In this case, advanced age (79 yrs), malnutrition (potassium at 3.10 mmol/L and albumin at 39.3 g/L), and specific anatomical regions (left pelvis) may be contributing factors to *Mycoplasma hominis* infections.

These findings underscore the importance of assessing individual risk factors, particularly in cases of culture-negative results. Clinicians should maintain a high index of suspicion for *Mycoplasma hominis* and other atypical pathogens in patients with delayed wound healing, unexpected infection courses, or those with known immunosuppressive conditions. It highlights the need to consider *Mycoplasma hominis is* in culture-negative wound infections to ensure timely and effective management. Improved diagnostic strategies and early recognition of at-risk individuals are critical to reducing morbidity and mortality associated with these rare but severe infections.

However, infections caused by *Mycoplasma hominis* remain underreported due to its slow growth and atypical presentation, often leading to diagnostic delays (9). Culturing Mycoplasma remains a trusted diagnostic approach for confirming infection. Advanced microbiological techniques, including mass pectrometry(MS) (33–35) and PCR (21, 36, 37), are crucial for identifying this pathogen. PCR is a molecular technique that amplifies specific DNA sequences, making it highly sensitive for the detection of *Mycoplasma hominis* (38). Studies have demonstrated that PCR exhibits a sensitivity of up to 90.5% for identifying *Mycoplasma hominis* in clinical specimens, ensuring reliable detection even at low pathogen concentrations (36)?. Furthermore, PCR achieves exceptional specificity, with reported rates as high as 99.2%, effectively minimizing false-positive results (36). However, its performance may be affected by the presence of inhibitors in clinical samples, which can reduce amplification efficiency and accuracy (39). Mass spectrometr(MS), particularly matrix-assisted laser desorption/ionization-time of flight (MALDI-TOF), has emerged as a rapid and effective method for identifying *Mycoplasma hominis* (33). Unlike PCR, MS does not require prior knowledge of the target organism, allowing for broad-spectrum pathogen identification (40). While MS provides rapid results, often within minutes, its sensitivity and specificity can be influenced by factors such as sample preparation and the comprehensiveness of the reference database (40). Moreover, MS generally requires viable organisms for accurate detection, which may limit its application in certain cases where sample viability is compromised (41).

The choice between PCR and MS for diagnosing *Mycoplasma hominis* infections should be guided by clinical context and resource availability. PCR is ideal for confirmatory diagnostics in suspected cases of *Mycoplasma hominis* infection due to its high sensitivity and specificity. Conversely, MS is better suited for rapid screening and identification of a broad range of pathogens in polymicrobial infections or when the causative organism is unknown. In practice, the integration of both methods may provide complementary benefits, improving diagnostic accuracy and efficiency in clinical microbiology. In this case, the patient’s sinus tract exudate was repeatedly tested for Mycobacterium to rule out tuberculosis infection. Gram staining of the bacterial smear showed no bacterial structures, and bacterial culture exhibited slow growth. Given these findings, we strongly suspected a mycoplasma infection. Mass spectrometry was subsequently used for identification, and confirmation with the mycoplasma IES kit confirmed the presence of *Mycoplasma hominis* infection.

Due to the lack of a cell wall, cell wall-targeting drugs like penicillins, cephalosporins, and vancomycin are ineffective (42, 43). Quinolones are considered the first-line drugs for treating mycoplasma infections, although resistance rates range from 15 to 30% (44). *Mycoplasma hominis* strains are highly sensitive to tetracyclines, lincosamides, and rifampin, which can be considered as first-choice medications (45). In a metadata of 26 studies conducted in 15 countries, Wen et al. reported the ratios of resistance to tetracycline, doxycycline, and minocycline in urogenital isolates of Mycoplasma and Ureaplasma were respectively reported as 14.2% (95% CI 8.2–23.2%), 5% (95% CI 3–8.1%), and 11.9% (95% CI 6.3–21.5%) (46).

In this case, the patient did not receive conventional antimycoplasma therapy instead of cefuroxime and penicillin sequentially, because her infectious symptoms had persisted for a month prior to admission. Prior to identifying the specific pathogen, empirical treatment with a second-generation cephalosporin was initiated based on the infection site and clinical presentation. However, the patient discontinued treatment and was discharged, which is why conventional antimycoplasma therapy was not administered. Interestingly, 1 month after discharge, the infected sinus tract achieved resolution following 1 week of penicillin treatment at a local clinic. The underlying mechanisms of this healing process warrant further investigation.

Prompt and precise diagnosis is critical for effective management and containment of Mycoplasma infections. Culturing Mycoplasma remains a trusted diagnostic approach for confirming infection. Clinically, *Mycoplasma hominis* infections are rare and easily misdiagnosed. Reliable detection is crucial for treating these infections. When pinpoint-sized colonies are observed on blood plates and Gram staining shows no bacteria, *Mycoplasma hominis* infection should be suspected. Clinical practitioners should recognize the potential for *Mycoplasma hominis* infections outside the urogenital tract. This case highlights the diagnostic challenges posed by *Mycoplasma hominis*. Traditional culture methods often fail due to its unique growth requirements, necessitating extended incubation periods. In this case, pinpoint like colonies were identified after 7 days of incubation and confirmed by mass spectrometry, emphasizing the importance of utilizing advanced diagnostic tools in suspected cases of atypical infections. Mycoplasma infection should be suspected when cell wall-targeting drugs are ineffective. For hard-to-culture microorganisms, extended culture times and the use of DNA sequencing, mass spectrometry, and biochemical reactions are recommended to aid in bacterial detection. Additionally, *Mycoplasma hominis* infection was effectively controlled under unconventional anti-mycoplasma therapy, and the underlying mechanisms require further investigation.

## 5 Limitations

First, the diagnosis of *Mycoplasma hominis* infection was based on culture and mass spectrometry, which despite being highly accurate, may not be widely accessible in many clinical settings, especially in resource-limited regions. The reliance on such advanced techniques limits the generalizability of the findings, as traditional diagnostic methods may delay detection. Additionally, while mass spectrometry provides rapid and specific identification, the sensitivity and specificity of this technique can vary depending on sample quality and preparation, which could potentially affect the diagnostic outcome in clinical practice.

Second, the patient’s treatment regimen, which included empirical antibiotic therapy before the specific pathogen was identified, raises questions regarding the effectiveness of such an approach in similar cases. Empirical treatment with broad-spectrum antibiotics may not always be sufficient, especially for infections caused by atypical organisms like *Mycoplasma hominis*. The delayed confirmation of *Mycoplasma hominis* infection and the use of non-standard antimicrobials in this case may limit the applicability of the results to other patients, particularly those with more severe or resistant infections.

Finally, the case presented is an isolated instance, and further studies involving the underlying mechanisms of infection healing in this patient are needed. In summary, the clinical management and treatment outcomes for *Mycoplasma hominis* infections in such cases remain an area requiring further exploration.

## Data Availability

The original contributions presented in this study are included in this article/supplementary material, further inquiries can be directed to the corresponding authors.

## References

[1] Razin S Yogev D Naot Y. Molecular biology and pathogenicity of mycoplasmas. *Microbiol Mol Biol Rev.* (1998) 62(4):1094–156. 9841667 10.1128/mmbr.62.4.1094-1156.1998 PMC98941

[2] Pereyre S Tardy F. Integrating the human and animal sides of Mycoplasmas resistance to antimicrobials. *Antibiotics (Basel).* (2021) 10(10):1216. 10.3390/antibiotics10101216 34680797 PMC8532757

[3] Taylor-Robinson D. Infections due to species of Mycoplasma and Ureaplasma: An update. *Clin Infect Dis.* (1996) 23(4):671–82. 8909826 10.1093/clinids/23.4.671

[4] Ljubin-Sternak S Mestrovic T. Chlamydia trachomatis and Genital Mycoplasmas: Pathogens with an impact on human reproductive health. *J Pathog.* (2014) 2014:183167. 10.1155/2014/183167 PMC4295611 25614838

[5] Mastromarino P Vitali B Mosca L. Bacterial vaginosis: A review on clinical trials with probiotics. *New Microbiol.* (2013) 36(3):229–38. 23912864

[6] Elias M Grzesko J Siejkowski R Nowicka J Maczynska B Goluda M [The presence of Mycoplasma hominis and Ureaplasma urealyticum in the cervical canal of uterus]. *Ginekol Pol.* (2005) 76(1):28–32. 15846863

[7] Bustos-Merlo A Rosales-Castillo A Cobo F Hidalgo-Tenorio C. Blood culture-negative infective endocarditis by Mycoplasma hominis: Case report and literature review. *J Clin Med.* (2022) 11(13):3841. 10.3390/jcm11133841 35807126 PMC9267468

[8] Contini C Rotondo J Magagnoli F Maritati M Seraceni S Graziano A Investigation on silent bacterial infections in specimens from pregnant women affected by spontaneous miscarriage. *J Cell Physiol.* (2018) 234(1):100–7. 10.1002/jcp.26952 30078192

[9] Stabler S Faure E Duployez C Wallet F Dessein R Le Guern R. The brief case: Mycoplasma hominis extragenital abscess. *J Clin Microbiol.* (2021) 59(4):e2343–2320. 10.1128/JCM.02343-20 PMC8092721 33826522

[10] Pereyre S Sirand-Pugnet P Beven L Charron A Renaudin H Barre A Life on arginine for Mycoplasma hominis: Clues from its minimal genome and comparison with other human urogenital mycoplasmas. *PLoS Genet.* (2009) 5(10):e1000677. 10.1371/journal.pgen.1000677 19816563 PMC2751442

[11] Evsyutina D Semashko T Galyamina M Kovalchuk S Ziganshin R Ladygina V Molecular basis of the slow growth of Mycoplasma hominis on different energy sources. *Front Cell Infect Microbiol.* (2022) 12:918557. 10.3389/fcimb.2022.918557 35873139 PMC9301678

[12] Pereyre SB Bebear C. *Mycoplasma Species Mycoplasma Hominis, Mycoplasma genitalium MfAwC.* Available online at: http://antimicrobe.org/m06.asp (accessed August 26, 2024).

[13] Waites K Katz B Schelonka R. Mycoplasmas and ureaplasmas as neonatal pathogens. *Clin Microbiol Rev.* (2005) 18(4):757–89. 16223956 10.1128/CMR.18.4.757-789.2005 PMC1265909

[14] Waites K Schelonka R Xiao L Grigsby P Novy M. Congenital and opportunistic infections: Ureaplasma species and Mycoplasma hominis. *Semin Fetal Neonatal Med.* (2009) 14(4):190–9. 10.1016/j.siny.2008.11.009 19109084

[15] Cazanave C Manhart L Bebear C. Mycoplasma genitalium, an emerging sexually transmitted pathogen. *Med Mal Infect.* (2012) 42(9):381–92. 22975074 10.1016/j.medmal.2012.05.006

[16] Plummer E Vodstrcil L Bodiyabadu K Murray G Doyle M Latimer R Are Mycoplasma hominis, Ureaplasma urealyticum and Ureaplasma parvum associated with specific genital symptoms and clinical signs in nonpregnant women? *Clin Infect Dis.* (2021) 73(4):659–68. 10.1093/cid/ciab061 33502501

[17] Moridi K Hemmaty M Azimian A Fallah M Khaneghahi Abyaneh H Ghazvini K. Epidemiology of genital infections caused by Mycoplasma hominis, M. genitalium and Ureaplasma urealyticum in Iran; A systematic review and meta-analysis study (2000–2019). *BMC Public Health.* (2020) 20(1):1020. 10.1186/s12889-020-08962-5 32600306 PMC7322857

[18] Razin S. The mycoplasmas. *Microbiol Rev.* (1978) 42(2):414–70. 353482 10.1128/mr.42.2.414-470.1978 PMC281436

[19] Nunez-Calonge R Caballero P Redondo C Baquero F Martinez-Ferrer M Meseguer M. Ureaplasma urealyticum reduces motility and induces membrane alterations in human spermatozoa. *Hum Reprod.* (1998) 13(1O):2756–61. 10.1093/humrep/13.10.2756 9804226

[20] Ahamad A Zervou F Aguero-Rosenfeld M. Extra-urogenital infection by Mycoplasma hominis in transplant patients: Two case reports and literature review. *BMC Infect Dis.* (2023) 23(1):601. 10.1186/s12879-023-08593-2 37710154 PMC10503128

[21] Sadeqi S Nikkhahi F Javadi A Eskandarion S Amin Marashi S. Development of multiplex real-time quantitative PCR for simultaneous detection of Chlamydia trachomatis, Mycoplasma hominis, Ureaplasma urealyticum, and Mycoplasma genitalium in infertile women. *Indian J Med Microbiol.* (2022) 40(2):231–4. 10.1016/j.ijmmb.2022.01.011 35144833

[22] Nulens E Van Praet J Selleslag D Van Landschoot T Dekeyzer D Descheemaecker P A disseminated Mycoplasma hominis infection in a patient with an underlying defect in humoral immunity. *Infection.* (2016) 44(3):379–81. 10.1007/s15010-015-0859-6 26546371

[23] Fernandez S Nicolas D Pericas J Castro Rebollo P Vila J Miro J A case of Mycoplasma hominis disseminated infection in a human immunodeficiency virus-1-infected pregnant woman with hypogammaglobulinemia. *J Microbiol Immunol Infect.* (2017) 50(1):118–9. 10.1016/j.jmii.2014.11.006 25648665

[24] Russo C Mikulska M Delfino E Toscanini F Mezzogori L Schiavoni R Mycoplasma hominis as cause of extragenital infection in patients with Hypogammaglobulinemia: Report of 2 cases and literature review. *Infect Dis Ther.* (2024) 13(10):2179–93. 10.1007/s40121-024-01035-9 39230828 PMC11416451

[25] Noska A Nasr R Williams D. Closed trauma, Mycoplasma hominis osteomyelitis, and the elusive diagnosis of Good’s syndrome. *BMJ Case Rep.* (2012) 2012:bcr2012007056. 10.1136/bcr-2012-007056 23188847 PMC4543976

[26] Tyner H Virk A Nassr A Razonable R. Mycoplasma hominis vertebral spine infection: Case report and a review of infections of bone and joints. *J Infect Chemother.* (2016) 22(11):755–8. 10.1016/j.jiac.2016.04.008 27234356

[27] Hulme-Jones J Gordon D Barbara J Li J. Mycoplasma hominis bursitis in a simultaneous pancreas-kidney transplant recipient: Case report and literature review. *Transpl Infect Dis.* (2020) 22(6):e13392. 10.1111/tid.13392 32603519

[28] Ruiz Castillo A Lopez Herrero E Tenorio Abreu A Gonzalez Gomez-Lozano A Saavedra Martin JM. Soft-tissue infection due to Mycoplasma hominis. *Rev Esp Quimioter.* (2023) 36(2):220–2. 36800686 10.37201/req/115.2022 PMC10066921

[29] Bethel J Tissingh E Vasireddy A Maxwell-Scott H. Mycoplasma hominis: Postoperative pelvic fracture-related infection in a trauma patient. *Br J Hosp Med (Lond).* (2022) 83(9):1–4. 10.12968/hmed.2022.0103 36193924

[30] Krijnen M Hekker T Algra J Wuisman P Van Royen B. Mycoplasma hominis deep wound infection after neuromuscular scoliosis surgery: The use of real-time polymerase chain reaction (PCR). *Eur Spine J.* (2006) 15(Suppl 5):599–603. 10.1007/s00586-005-0055-y 16429284 PMC1602191

[31] Pigrau C Almirante B Gasser I Pahissa A. Sternotomy infection due to Mycoplasma hominis and Ureaplasma urealyticum. *Eur J Clin Microbiol Infect Dis.* (1995) 14(7):597–8. 7588844 10.1007/BF01690731

[32] Garcia-de-la-Fuente C Minambres E Ugalde E Saez A Martinez-Martinez L Farinas M. Post-operative mediastinitis, pleuritis and pericarditis due to Mycoplasma hominis and Ureaplasma urealyticum with a fatal outcome. *J Med Microbiol.* (2008) 57(Pt 5):656–7. 10.1099/jmm.0.47632-0 18436601

[33] Su F Zhang J Zhu Y Lv H Ge Y. Identification of sacrococcygeal and pelvic abscesses infected with invasive Mycoplasma hominis by MALDI-TOF MS. *J Clin Lab Anal.* (2022) 36(4):e24329. 10.1002/jcla.24329 35285086 PMC8993641

[34] Pailhories H Rabier V Eveillard M Mahaza C Joly-Guillou M Chennebault J A case report of Mycoplasma hominis brain abscess identified by MALDI-TOF mass spectrometry. *Int J Infect Dis.* (2014) 29:166–8. 10.1016/j.ijid.2014.08.004 25449252

[35] Kitagawa H Shimizu H Katayama K Tadera K Nomura T Omori K Postoperative mediastinitis after cardiac surgery caused by Mycoplasma hominis: A case report. *Surg Case Rep.* (2021) 7(1):248. 34812956 10.1186/s40792-021-01326-0 PMC8611127

[36] Cunningham S Mandrekar J Rosenblatt J Patel R. Rapid PCR Detection of Mycoplasma hominis, Ureaplasma urealyticum, and Ureaplasma parvum. *Int J Bacteriol.* (2013) 2013:168742. 10.1155/2013/168742 PMC4745450 26904723

[37] Carneiro F Daros A Daros A de Castro T de Vasconcelos Carneiro M Fidelis C Cervical cytology of samples with Ureaplasma urealyticum, Ureaplasma parvum, Chlamydia trachomatis, Trichomonas vaginalis, Mycoplasma hominis, and *Neisseria gonorrhoeae* detected by Multiplex PCR. *Biomed Res Int.* (2020) 2020:7045217. 10.1155/2020/7045217 32724807 PMC7366191

[38] Grau O Kovacic R Griffais R Launay V Montagnier L. Development of PCR-based assays for the detection of two human mollicute species, Mycoplasma penetrans and M. hominis. *Mol Cell Probes.* (1994) 8(2):139–47. 10.1006/mcpr.1994.1019 7935512

[39] Trombley Hall A McKay Zovanyi A Christensen D Koehler J Devins Minogue T. Evaluation of inhibitor-resistant real-time PCR methods for diagnostics in clinical and environmental samples. *PLoS One.* (2013) 8(9):e73845. 10.1371/journal.pone.0073845 24040090 PMC3767612

[40] Haider A Ringer M Kotroczó Z Mohácsi-Farkas C Kocsis T. The current level of MALDI-TOF MS applications in the detection of microorganisms: A short review of benefits and limitations. *Microbiol Res.* (2023) 14(1):80–90.

[41] Calderaro A Chezzi C. MALDI-TOFMS: A reliable tool in the real life of the clinical microbiology laboratory. *Microorganisms.* (2024) 12(2):322. 10.3390/microorganisms12020322 38399726 PMC10892259

[42] Wang H Ren D Li H Wang S. Periprosthetic joint infection caused by Mycoplasma hominis, diagnosed using metagenomic sequencing. *Int J Gen Med.* (2021) 14:7003–6. 10.2147/IJGM.S330924 34707391 PMC8544117

[43] Ahmed J Rawre J Dhawan N Khanna N Dhawan B. Mycoplasma hominis: An under recognized pathogen. *Indian J Med Microbiol.* (2021) 39(1):88–97. 33610259 10.1016/j.ijmmb.2020.10.020

[44] Ahn J Cho H Li D Choi M Lee J Eun B Efficacy of tetracyclines and fluoroquinolones for the treatment of macrolide-refractory Mycoplasma pneumoniae pneumonia in children: A systematic review and meta-analysis. *BMC Infect Dis.* (2021) 21(1):1003. 10.1186/s12879-021-06508-7 34563128 PMC8465761

[45] Dawood A Algharib S Zhao G Zhu T Qi M Delai K Mycoplasmas as host pantropic and specific pathogens: Clinical implications, gene transfer, virulence factors, and future perspectives. *Front Cell Infect Microbiol.* (2022) 12:855731. 10.3389/fcimb.2022.855731 35646746 PMC9137434

[46] Wen X Nobakht M Yang Y Kouhsari E Hajilari S Shakourzadeh M Tetracyclines resistance in Mycoplasma and Ureaplasma urogenital isolates derived from human: A systematic review and meta-analysis. *Ann Clin Microbiol Antimicrob.* (2023) 22(1):83. 10.1186/s12941-023-00628-5 37697380 PMC10496389

[47] Sielaff T Everett J Shumway S Wahoff D Bolman R III Dunn D. Mycoplasma hominis infections occurring in cardiovascular surgical patients. *Ann Thorac Surg.* (1996) 61(1):99–103. 8561647 10.1016/0003-4975(95)00826-8

[48] Mossad S Rehm S Tomford J Isada C Taylor P Rutherford I Sternotomy infection with Mycoplasma hominis: A cause of “culture negative” wound infection. *J Cardiovasc Surg (Torino).* (1996) 37(5):505–9. 8941693

[49] Vogel U Luneberg E Kuse E Neulinger A Frosch M. Extragenital Mycoplasma hominis infection in two liver transplant recipients. *Clin Infect Dis.* (1997) 24(3):512–3. 10.1093/clinids/24.3.512 9114209

[50] Hopkins P Winlaw D Chhajed P Harkness J Horton M Keogh A Mycoplasma hominis infection in heart and lung transplantation. *J Heart Lung Transplant.* (2002) 21(11):1225–9. 12431497 10.1016/s1053-2498(02)00427-8

[51] Marini H Merle V Frebourg N Godier S Bastit D Benadiba L Mycoplasma hominis wound infection after a vascular allograft. *J Infect.* (2008) 57(3):272–4. 10.1016/j.jinf.2008.06.003 18649944

[52] Lee J Lee J Lee N Ha C Chung D Peck K. [Two cases of septic arthritis by Mycoplasma hominis after total knee replacement arthroplasty]. *Korean J Lab Med.* (2009) 29(2):135–9. 10.3343/kjlm.2009.29.2.135 19411780

[53] Myers P Khabiri E Greub G Kalangos A. Mycoplasma hominis mediastinitis after acute aortic dissection repair. *Interact Cardiovasc Thorac Surg.* (2010) 11(6):857–8. 10.1510/icvts.2010.244608 20826555

[54] Yamakami S Mikami Y Watanabe K Saya Y Tanaka C Eto H [A case of mycoplasma hominis infection on chronic refractory lower leg ulceration caused by livedo vasculopathy]. *Rinsho Byori.* (2012) 60(11):1040–4. 23383571

[55] Flouzat-Lachaniette C Guidon J Allain J Poignard A. An uncommon case of Mycoplasma hominis infection after total disc replacement. *Eur Spine J.* (2013) 22(Suppl 3):S394–8. 10.1007/s00586-012-2511-9 23001380 PMC3641271

[56] Wynes J Harris WT Hadfield RA Malay DS. Subtalar joint septic arthritis in a patient with hypogammaglobulinemia. *J Foot Ankle Surg.* (2013) 52(2):242–8. 10.1053/j.jfas.2012.10.012 23153784

[57] Whitson W Ball P Lollis S Balkman J Bauer D. Postoperative Mycoplasma hominis infections after neurosurgical intervention. *J Neurosurg Pediatr.* (2014) 14(2):212–8. 24856879 10.3171/2014.4.PEDS13547

[58] Antonic M Djordjevic A Juric P Pirnat M Gorisek Miksic N. Mycoplasma hominis ascending aortic graft infection successfully treated with graft preservation using negative pressure wound therapy with instillation and dwell time. *Wounds.* (2020) 32(12):E67–70. 33476287

[59] Ng S Kumar S Loo W. Mycoplasma hominis lumbar wound infection after posterior decompression and instrumented fusion: A case report. *JBJS Case Connect.* (2021) 11(2). 10.2106/JBJS.CC.20.00439 33950867

[60] Qamar Z Tjoumakaris S Pattengill M Ahmed M Hess B. Intracranial Mycoplasma hominis infection following emergent craniectomy. *IDCases.* (2021) 25:e01175. 10.1016/j.idcr.2021.e01175 34159053 PMC8203728

[61] Huang S Tang Y Wang J Wang X Zhang Y Pan S. Case Report: Double trouble: A rare case of successfully treated Mycoplasma hominis and *Pseudomonas aeruginosa* co-infection. *Front Cell Infect Microbiol.* (2023) 13:1159891. 10.3389/fcimb.2023.1159891 37197207 PMC10183579

[62] Kuo L Tseng Y Chen L Wang T Chen C Lee H. Infection of Mycoplasma hominis in the left lower leg amputation wound of a patient with diabetes: A case report. *J Med Case Rep.* (2024) 18(1):380. 10.1186/s13256-024-04718-6 39143557 PMC11325761

